# Factors associated with diarrhea in children under five years old in the state of Pernambuco, according to surveys conducted in 1997 and 2006

**DOI:** 10.11606/S1518-8787.2018052016094

**Published:** 2018-04-20

**Authors:** Maria Josemere de Oliveira Borba Vasconcelos, Anete Rissin, José Natal Figueiroa, Pedro Israel Cabral de Lira, Malaquias Batista

**Affiliations:** I Instituto de Medicina Integral Prof. Fernando Figueira. Diretoria de Pesquisas. Recife, PE, Brasil; II Universidade Federal de Pernambuco. Departamento de Nutrição. Recife, PE, Brasil

**Keywords:** Child, Preschool, Diarrhea, Infantile, epidemiology, Diarrhea, epidemiology, Prevalence, Risk Factors, Socioeconomic Factors, Time Series Studies, Pré-Escolar, Diarreia Infantil, epidemiologia, Diarreia, epidemiologia, Prevalência, Fatores de Risco, Fatores Socioeconômicos, Estudos de Séries Temporais

## Abstract

**OBJECTIVE:**

Describe and compare variations of the factors associated with the prevalence of diarrhea in children under five years old in the state of Pernambuco.

**METHODS:**

We used the databases of two population-based surveys from the years 1997 and 2006, with 2,078 and 1,650 children, respectively, evaluated in 18 municipalities of Pernambuco (Metropolitan Region of Recife, urban and rural interior). The variables, allocated at hierarchical levels, were analyzed using prevalence and Poisson regression ratios.

**RESULTS:**

Only four variables were independently associated and were included in the final hierarchical model: geographical area, number of people per room, maternal age and the age of the child. In 1997: urban interior = 1.33 (95%CI 1.06–1.66), rural interior = 1.22 (95%CI 0.97–1.53) and in 2006: urban interior = 1.87 (95%CI 1.31–2.66), rural interior = 2.07 (95%CI 1.50–2.85); number of persons per room (1997): 1 to less than 2 = 1.29 (95%CI 0.98–1.68), two or more = 1.47 (95%CI 1.11–1.95) and in 2006: 1 to less than 2 = 0.86 (95%CI 0.68–1.09), two or more = 1.29 (95%CI 0.94–1.75); maternal age (1997): 10 to 19 years = 1.48 (95%CI 1.05–2.08), 20 to 24 years = 1.23 (95%CI 0.94–1.60), 25 to 34 years = 1.01 (95%CI 0.78–1.30) and in 2006: 10 to 19 years old = 1.70 (95%CI 1.08–2.66), 20 to 24 years old = 1.64 (95%CI 1.16–2.32), 25 to 34 years = 1.20 (95%CI 0.89–1.62); and age of the child (1997): 0–11 months = 1.57 (95%CI 1.27–1.94), 12–23 months = 1.73 (95%CI 1.41–2.12) and in 2006: 0–11 months = 1.04 (95%CI 0.76–1.41), 12–23 months = 1.77 (95%CI 1.41–2.23).

**CONCLUSIONS:**

There was a great variability of the conditioners of diarrhea in children between the two periods analyzed. At the public policy level, despite changes in terms of people, time sequences, and geographic spaces, diarrhea remains on an important scale in the ranking of government power.

## RESUMO

**OBJETIVO:**

Descrever e comparar variações dos fatores associados à prevalência de diarreia em menores de cinco anos no estado de Pernambuco.

**MÉTODOS:**

Foram utilizados os bancos de dados de dois inquéritos de base populacional nos anos de 1997 e 2006, com 2.078 e 1.650 crianças, respectivamente, avaliadas em 18 municípios de Pernambuco (Região Metropolitana do Recife, interior urbano e rural). As variáveis, alocadas em níveis hierárquicos, foram analisadas por meio de razões de prevalência e regressão de Poisson.

**RESULTADOS:**

Apenas quatro variáveis se mostraram independentemente associadas e constaram no modelo final hierarquizado: área geográfica, número de pessoas por cômodo, idade materna e idade da criança. Em 1997: interior urbano = 1,33 (IC95% 1,06–1,66), interior rural = 1,22 (IC95% 0,97–1,53) e em 2006: interior urbano = 1,87 (IC95% 1,31–2,66), interior rural = 2.07 (IC95% 1.50–2.85); número de pessoas por cômodo (1997): 1 a menos de 2 = 1,29 (IC95% 0,98-1,68), dois ou mais = 1,47 (IC95% 1,11–1,95) e em 2006: 1 a menos de 2 = 0.86 (IC95% 0,68–1,09), dois ou mais = 1,29 (IC95% 0,94–1,75); idade materna (1997): 10 a 19 anos = 1,48 (IC95% 1,05–2,08), 20 a 24 anos = 1,23 (IC95% 0,94–1,60), 25 a 34 anos = 1,01 (IC95% 0,78–1,30) e em 2006: 10 a 19 anos = 1,70 (IC95% 1,08–2,66), 20 a 24 anos = 1,64 (IC95% 1,16–2,32), 25 a 34 anos = 1,20 (IC95% 0,89–1,62); e idade da criança (1997): 0–11 meses = 1,57 (IC95% 1,27–1,94), 12–23 meses = 1,73 (IC95% 1,41–2,12) e em 2006: 0–11 meses = 1,04 (IC95% 0,76–1,41), 12–23 meses = 1,77 (IC95% 1,41–2,23).

**CONCLUSÕES:**

Houve uma grande variabilidade dos condicionantes das diarreias em crianças entre os dois períodos analisados. Em nível de políticas públicas, apesar de mudanças em termos de pessoas, sequências temporais e espaços geográficos, as diarreias continuam em uma escala importante no elenco de prioridades do poder governamental.

## INTRODUCTION

The diarrheal disease continues to be one of the main problems affecting the infant population in the first years of life, especially in less developed regions. Due to its high morbidity and mortality, it is a priority public health issue^1^ ^,^ ^2^ and represents an important demand in the health services network at a global, regional and local scale^3^ ^,^ ^4^. In several countries, diarrhea still appears as the main cause of infant death, consisting of a discriminatory indicator of geographic spaces characterized by precarious conditions of collective life, typifying the so-called poverty ecosystems^1^ ^,^ ^5^.

The problem is related to environmental, socioeconomic and cultural factors, low coverage and effectiveness of health services. These factors can establish marked differences in their evolution due to the inequalities that compromise the profile of production and distribution of goods and services in the context of different strata of the population^6^ ^,^ ^7^.

Due to their clinical and epidemiological characteristics, diarrheas may appear as “tracer conditions”^8^, since the descriptive and analytical monitoring of their temporal and spatial evolution, rather than the demarcation of a specific nosology, may represent the cartography of the underdevelopment and poverty, characterized mainly by the occurrence of preventable and curable diseases.

These conceptual and empirical aspects justify the interest in continuous or periodic evaluations on the historical, geographical trends and their risk factors, to represent the degree of human development of the population and health services and actions from a territorial and temporal perspective. From the epidemiological point of view, the configuration of the factor complexes significantly correlated with its occurrence – including intermediate events such as hospitalization, or final ones, such as specific mortality – provides a framework of important benchmarks for the policy and programmatic challenges of the health sector.

The study of periodic population-based studies in the state of Pernambuco on maternal and child health problems provides, in particular, the evaluation of the profound changes in morbidity profiles since 1990 when the most dynamic process of the so-called epidemiological transition^9^ ^,^ ^10^ is formed. Based on the initial study (1991) on maternal and child health, the prevalence of diarrhea (22.5%) in children in Pernambuco was already much higher than in the states of the Northeast (14.4%) and the Southeast (8%)^11^. This approach stands out when one incorporates a multifactorial view of the context, i.e., a holistic perspective of health^12^. It is pertinent to consider that another population-based assessment of the problems and demands of health services in the state of Pernambuco, the IV PESN, will soon be carried out in order to update information on the evolution of various maternal and child health problems, including on the behavior of diarrhea and its participation in the composition of outpatient demands, hospitalizations and major causes of death.

The objective of this study is to describe and compare variations of factors associated with diarrhea in children under five years of age in the state of Pernambuco. To this end, a descriptive and analytical inventory of diarrheal diseases in children from that state was carried out, based on two field surveys conducted in 1997 and 2006, as part of the wider institutional project that should be to focus on spatial and temporal trends in the occurrence of the problem, as well as to determine its possible risk factors.

## METHODS

This cross-sectional population-based study used secondary data from the State Health and Nutrition Surveys (II and III PESN) II and III, carried out in 1997 and 2006, representing the urban strata (Metropolitan Region of Recife and urban interior [UI]) and rural (rural interior [RI]), aiming at updating and expanding the diagnosis of health, nutrition, food, service demands and socioeconomic conditions of the population of the state of Pernambuco^10^ ^,^ ^13^.

The study population was constituted by children under five, included in the two surveys mentioned above, with 2,078 and 1,650 children, evaluated in 1997 and 2006, respectively.

The sampling process of these surveys was probabilistic and stratified in three stages. Initially, it consisted of a lottery of the municipalities, then the census tracts and finally of the households. The 18 municipalities included in the PESN II and III were: Recife, Olinda, Paulista, Jaboatão, Cabo, São Bento do Una, Goiana, Itaíba, Belém do São Francisco, Orobó, Caruaru, Camocim de São Félix, Triunfo, Bodocó, Palmares, Ribeirão, Panelas, and Itaquitinga.

The interviews were carried out with the mother or the caretaker, applying forms and questionnaires composed of pre-coded questions containing socioeconomic, environmental, demographic, biological information, maternal characteristics and access to health services. The dependent variable was represented by diarrhea morbidity, referenced in the last two weeks prior to the study. The occurrence of three or more daily bowel movements, of liquid or semi-liquid consistency, with or without mucus or blood^14^, was defined as the case.

The independent variables were grouped into: a) geographic factor (urban and rural); b) environmental factors (water supply, sewage treatment, drinking water treatment, waste disposal, type of floor, type of wall, number of people per room and number of people per bedroom); c) socioeconomic factors (possession of a refrigerator, visits by the community health agent and *per capita* family income); d) maternal factors (age, work condition, and schooling); e) biological factors (birth weight, nutritional status and breastfeeding).

The nutritional status of the children was analyzed by means of anthropometry, using the indicators weight for age, height for age and weight for height, according to the distribution of Z scores. For the classification of anthropometric data, the reference standard adopted by the Ministry of Health^15^ was chosen, using the software Anthro, version 3.2.2^16^.

To identify factors associated with the occurrence of diarrhea, both in 1997 and in 2006, the predictor variables were hierarchized at three levels by conceptual logic criteria (Figure). The construction of the hierarchical model used in this study had as reference the modeling applied by Fuchs et al. for the investigation of risk factors for severe diarrhea^17^. As control variables, the variables sex and age were selected, which, because of their biological meaning and without receiving influence from other variables, could directly interfere in the determination of the investigated problem. From this disposition by levels, we used a univariate analysis process by grouping the categories of variables in each group by calculating prevalence ratios (PR) and respective confidence intervals (95%CI) and adopting p values below 0.25 as of statistical significance. The variables with this value were allowed to integrate the sequence of multivariate analyzes (Poisson regression), starting from the most distal level (geographical categories) to the most proximal ones (variables referring to the child). At each of the following levels, the Poisson multiple regression model was adjusted with the variables of the corresponding level and statistically significant variables of the previous groups. The final model was composed of the variables of the level that presented p-value < 0.05, as well as by the other variables of the previous levels in the same condition. The model adjustments considered the effect of clusters present in the data. The purposeful selection strategy was used to choose the explanatory variables of the models at each level. Poisson regression analysis was performed using Stata 12.1SE software.

**Figure:**
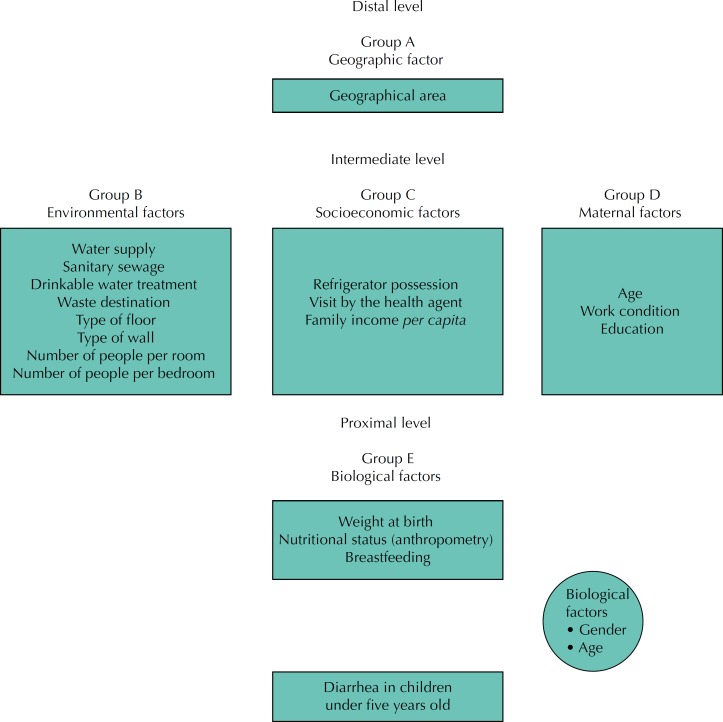
A hierarchical causal model of the possible factors associated with diarrhea in children under five years old in the state of Pernambuco in 1997 and 2006.

It should be noted that the rotavirus vaccine began to be applied (children) at the beginning of the last field survey (March to May) so that its impact would not yet be detectable at the population level.

The surveys (II and III PESN) were approved by the Human Research Ethics Committee of the Centro de Ciências da Saúde da Universidade Federal de Pernambuco (II PESN – 2/27/1997) and the Instituto de Medicina Integral Professor Fernando Figueira (III PESN – 11/9/2005).

## RESULTS

The prevalence of diarrhea dropped from 19.8% (1997) to 18.1% (2006). In relation to Group A (geographic factors), the notable reduction in the Metropolitan Region of Recife, from 16.9% to 10.5%, was notable. As a statistically significant association source with diarrhea, we found that the geographical strata were presented in the two surveys, with the lowest risks in the Metropolitan Region of Recife (Table 1). In Group B (environmental factors), composed of eight variables, sanitary sewage, waste destination, number of people per room and treatment of drinking water were common risk factors in the years analyzed. The public water supply, as well as the treatment of drinking water, were statistically significant only in the univariate analyzes for the year 2006. In Group C (socioeconomic factors), the possession of a refrigerator is a protective factor for the occurrence of diarrhea in 1997 and 2006, in the same way as the *per capita* family income. Among the variables included in Group D (maternal factors), low maternal age and schooling behaved as risk factors for diarrhea in their children, in the two years evaluated.

**Table 1:** Univariate analysis of the factors associated with the occurrence of diarrhea in children under five years of age. State of Pernambuco, Brazil, 1997 and 2006.

Variable			1997				2006			
			Sample	Diarrhea	PR (95%CI)	p	Sample	Diarrhea	PR (95%CI)	p
			N	n (%)			N	n (%)		
Distal level										
Group A										
	Geographical area									
		Metropolitan region	734	124 (16.9)	1.0	0.043	427	45 (10.5)	1.0	< 0.001
		Urban interior	685	154 (22.5)	1.33 (1.06–1.66)		416	81 (19.5)	1.87 (1.31–2.66)	
		Rural interior	651	131 (20.1)	1.22 (0.97–1.53)		789	169 (21.4)	2.07 (1.50–2.85)	
Intermediate level										
Group B										
	Water supply									
		General network	1,359	260 (19.1)	1.0	0.243	918	148 (16.1)	1.0	0.018
		Other	711	149 (21.0)	1.12 (0.93–1.35)		714	147 (20.6)	1.29 (1.04–1.60)	
	Sanitary sewage									
		Public network	554	95 (17.1)	1.0	0.004	547	83 (15.2)	1.0	0.045
		Septic tank covers	724	130 (18.0)	1.05 (0.82–1.35)		519	93 (17.9)	1.18 (0.90–1.56)	
		Other	792	184 (23.2)	1.40 (1.11–1.76)		563	119 (21.1)	1.39 (1.07–1.81)	
	Drinkable water treatment									
		General network	1,207	209 (17.3)	1.0	< 0.001	823	104 (12.6)	1.0	< 0.001
		Other	863	200 (23.2)	1.34 (1.12–1.61)		809	191 (23.6)	1.78 (1.35–2.35)	
	Waste destination									
		Public waste collection	1,046	177 (16.9)	1.0	< 0.001	945	151 (16.0)	1.0	0.008
		Others	1,024	232 (22.7)	1.37 (1.14–1.65)		687	144 (21.0)	1.33 (1.08–1.65)	
	Type of floor									
		Ceramics/Cement	1,780	343 (19.3)	1.0	0.183	1,544	277 (17.9)	1.0	0.537
		Others	290	66 (22.8)	1.18 (0.92–1.51)		88	18 (20.4)	1.14 (0.75–1.74)	
	Type of wall									
		Masonry/Brick	1,771	347 (19.6)	1.0	0.544	1,525	273 (17.9)	1.0	0.550
		Other	299	62 (20.7)	1.08 (0.84–1.39)		107	22 (20.6)	1.14 (0.75–1.72)	
	No. of people per room									
		< 1	429	63 (14.7)	1.0	0.004	653	113 (17.3)	1.0	0.015
		1–2	1,014	200 (19.7)	1.34 (1.03–1.75)		780	132 (16.9)	0.96 (0.76–1.21)	
		≥ 2	627	146 (23.3)	1.59 (1.21–2.10)		199	50 (25.1)	1.45 (1.08–1.95)	
	No. of people per bedroom									
		< 2	364	54 (14.8)	1.0	0.022	334	52 (15.6)	1.0	0.405
		2–3	675	131 (19.4)	1.31 (0.97–1.75)		599	108 (18.0)	1.15 (0.85–1.57)	
		≥ 3	1,031	224 (21.7)	1.46 (1.11–1.92)		699	135 (19.3)	1.23 (0.91–1.65)	
Group C										
	Refrigerator possession									
		Yes	1,138	178 (15.6)	1.0	< 0.001	1,105	179 (16.2)	1.0	0.007
		No	932	231 (24.8)	1.58 (1.32–1.90)		527	116 (22.0)	1.35 (1.09–1.68)	
	Visit by the health agent									
		Yes	680	151 (22.2)	1.0	0.081	1,353	248 (18.3)	1.0	0.672
		No	1,377	256 (18.6)	0.85 (0.70–1.02)		276	47 (17.0)	0.94 (0.71–1.24)	
	Family income *per capita* (MW)									
		< 0.50	1,253	285 (22.7)	1.50 (1.22–1.83)	< 0.001	1,366	267 (19.5)	2.18 (1.44–3.32)	< 0.001
		≥ 0.50	798	122 (15.3)	1.0		233	21 (9.0)	1.0	
Group D										
	Maternal age (years)									
		10–19	170	47 (27.6)	1.58 (1.12–2.23)	0.002	100	24 (24.0)	1.64 (1.06–2.52)	0.009
		20–24	568	131 (23.1)	1.31 (1.00–1.72)		363	83 (22.9)	1.57 (1.15–2.14)	
		25–34	922	159 (17.2)	0.97 (0.75–1.26)		795	134 (16.9)	1.15 (0.86–1.55)	
		≥ 35	408	72 (17.6)	1.0		371	54 (14.6)	1.0	
	Mother's work condition									
		Is employed	610	98 (16.1)	1.0	0.010	132	24 (18.2)	1.0	0.923
		Is unemployed	1,456	310 (21.3)	1.32 (1.07–1.64)		1,496	270 (18.0)	0.98 (0.67–1.43)	
	Maternal education (years)									
		< 4	863	205 (23.8)	2.05 (1.57–2.67)	< 0.001	524	110 (21.0)	1.47 (1.11–1.94)	0.028
		4–7	644	138 (2.14)	1.85 (1.40–2.44)		623	117 (18.8)	1.31 (0.99–1.74)	
		≥ 8	547	63 (11.5)	1.0		474	67 (14.1)	1.0	
Proximal level										
Group E										
	Child's age (months)									
		0–11	458	114 (24.9)	1.63 (1.32–2.01)	< 0.001	305	52 (17.0)	1.14 (0.85–1.53)	< 0.001
		12–23	413	112 (27.1)	1.77 (1.45–2.17)		356	100 (28.1)	1.92 (1.54–2.41)	
		24–59	1,199	183 (1.53)	1.0		971	143 (14.7)	1.0	
	Gender									
		Male	1,028	206 (20.0)	1.03 (0.87–1.23)	0.725	841	161 (19.1)	1.13 (0.92–1.40)	0.251
		Female	1,042	203 (19.5)	1.0		791	134 (16.9)	1.0	
	Weight at birth (gram)									
		< 2.500	150	36 (24.0)	1.14 (0.83–1.56)	0.682	138	20 (14.5)	0.80 (0.52–1.22)	0.503
		2.500–2.999	373	74 (19.8)	0.98 (0.77–1.23)		294	57 (19.4)	1.06 (0.81–1.39)	
		≥ 3,000	1,377	282 (20.5)	1.0		1,148	207 (18.0)	1.0	
	Height for age									
		Very low/Low	238	64 (26.9)	1.38 (1.09–1.75)	0.007	138	31 (22.5)	1.24 (0.89–1.73)	0.195
		Adequate	1,757	335 (19.1)	1.0		1,445	257 (17.8)	1.0	
	Weight for height									
		Accentuated thinness/Thinness	36	6 (16.7)	0.92 (0.47–1.81)	0.007	25	4 (16.0)	0.84 (0.36–2.00)	0.303
		Eutrophy	1,473	268 (18.2)	1.0		1,092	207 (19.0)	1.0	
		Risk of overweight/Overweight/Obesity	512	127 (24.8)	1.35 (1.12–1.62)		467	75 (16.1)	0.82 (0.64–1.06)	
	Weight for age									
		Very low/Low	100	23 (23.0)	1.19 (0.81–1.74)	0.380	51	14 (27.5)	1.48 (0.92–2.38)	0.103
		Adequate/High	1,933	381 (19.7)	1.0		1,540	275 (17.9)	1.0	
	Breastfeeding									
		Is/was breastfed	1,840	363 (19.7)	1.0	0.886	1,542	279 (18.1)	1.0	0.903
		Never breastfed	215	42 (19.5)	0.98 (0.73–1.32)		81	15 (18.5)	1.03 (0.64–1.65)	

MW: minimum wage

The results of the hierarchical final model for the factors associated with the occurrence of diarrhea are shown in Table 2. Of a total of 10 variables, only four were independently associated: geographical area, number of people per room, maternal age and age of the child. The results are presented below, with the respective prevalence ratios and confidence intervals. In 1997: UI = 1.33 (95%CI 1.06–1.66), RI = 1.22 (95%CI 0.97–1.53) and occurrence of diarrhea (2006): UI = 1.87 (95%CI 1.31–2.66), RI(2006) = 2.07 (95%CI 1.50–2.85); number of persons per room (1997): 1 to less than 2 = 1.29 (95%CI 0.98–1.68), two or more = 1.47 (95%CI 1.11–1.95) and number of people per room (2006): 1 to less than 2 = 0.86 (95%CI 0.68–1.09), two or more = 1.29 (95%CI 0.94–1.75); maternal age (1997): 10 to 19 years = 1.48 (95%CI 1.05–2.08), 20 to 24 years = 1.23 (95%CI 0.94–1.60), 25 to 34 years = 1.01 (95%CI 0,78–1,30) and maternal age (2006): 10 to 19 years old = 1.70 (95%CI 1.08–2.66), 20 to 24 years old = 1.64 (95%CI 1.16–2.32), 25 to 34 years = 1.20 (95%CI 0.89–1.62); and age of the child (1997): 0–11 months = 1.57 (95%CI 1.27–1.94), 12–23 months = 1.73 (95%CI 1.41–2.12) and the age of the child (2006): 0–11 months = 1.04 (95%CI 0.76–1.41), 12–23 months = 1.77 (95%CI 1.41–2.23).

**Table 2:** Hierarchical final models of diarrhea in children under five years of age. State of Pernambuco, Brazil, 1997 and 2006.

Variable			1997				2006			
			Sample	Diarrhea	PR (95%CI)^a^	p	Sample	Diarrhea	PR (95%CI)^a^	p
			N	n (%)			N	n (%)		
Distal level										
Group A										
	Geographical area									
		Metropolitan region	734	124 (16.9)	1.0	0.043	427	45 (10.5)	1.0	< 0.001
		Urban interior	685	154 (22.5)	1.33 (1.06–1.66)		416	81 (19.5)	1.87 (1.31–2.66)	
		Rural interior	651	131 (20.1)	1.22 (0.97–1.53)		789	169 (21.4)	2.07 (1.50–2.85)	
Intermediate level										
Group B										
	Waste destination									
		Public waste collection	1,046	177 (16.9)	1.0	< 0.001^b^	-	-	-	-
		Others	1,024	232 (22.7)	1.50 (1.19–1.88)		-	-	-	-
	No. of people per room									
		< 1	429	63 (14.7)	1.0	0.026^b^	653	113 (17.3)	1.0	
		1–2	1,014	200 (19.7)	1.29 (0.98–1.68)		780	132 (16.9)	0.86 (0.68–1.09)	0.028
		≥ 2	627	146 (23.3)	1.47 (1.11–1.95)		199	50 (25.1)	1.29 (0.94–1.75)	
	Water supply									
		General network	-	-	-	-	918	148 (16.1)	1.0	0.028
		Others	-	-	-	-	714	147 (20.6)	0.67 (0.47–0.96)	
	Drinkable water treatment									
		Boiled/Filtered/Chlorinated Mineral	-	-	-	-	823	104 (12.6)	1.0	< 0.001
		Strained/Untreated/Other	-	-	-	-	809	191 (23.6)	1.77 (1.35–2.32)	
Distal level										
Group C										
	Refrigerator possession									
		Yes	1,138	178 (15.6)	1.0	0.001^c^	-	-	-	-
		No	932	231 (24.8)	1.45 (1.16–1.80)		-	-	-	-
	*Per capita* income									
		< 0.50	-	-	-	-	1,366	267 (19.5)	1.67 (1.06–2.61)	0.026
		≥ 0.50	-	-	-	-	233	21 (9.0)	1.0	
Group D										
	Maternal age (years)									
		10–19	169	47 (27.8)	1.48 (1.05–2.08)	0.037^d^	98	24 (24.5)	1.70 (1.08–2.66)	0.014^d^
		20–24	561	129 (23.0)	1.23 (0.94–1.60)		344	78 (22.7)	1.64 (1.16–2.32)	
		25–34	919	158 (17.2)	1.01 (0.78–1.30)		780	131 (16.8)	1.20 (0.89–1.62)	
		≥ 35	403	72 (17.9)	1.0		365	53 (14.5)	1.0	
	Maternal education (years)									
		< 4	861	205 (23.8)	1.65 (1.21–2.27)	0.006^e^	-	-	-	-
		4–7	644	138 (21.4)	1.55 (1.15–2.09)		-	-	-	-
		≥ 8	547	63 (11.5)	1.0		-	-	-	-
Proximal level										
Group E										
	Child's age (months)									
		0–11	454	113 (24.9)	1.57 (1.27–1.94)		296	49 (16.6)	1.04 (0.76–1.41)	
		12–23	410	112 (27.3)	1.73 (1.41–2.12)	< 0.001^f^	347	97 (28.0)	1.77 (1.41–2.23)	< 0.001^f^
		24–59	1,188	181 (15.2)	1.0		953	142 (14.9)	1.0	

a Razões de prevalências ajustadas.

b Ajustados para a variável do nível A.

c Ajustado para as variáveis dos níveis A e B.

d Ajustados para as variáveis dos níveis A, B e C.

e Ajustado para as variáveis dos níveis A, B, C e D.

f Ajustado para as variáveis dos níveis A, B, C, D e E.

## DISCUSSION

Health problems, when considered at the population level and as individual or clinical processes, involve multiple factors in their determination. Thus, the variations occurring in the geographical space between biological and social groups and, especially, on a temporal scale, such as the historical trends of the health or disease process, represent multifactorial outcomes, combining a more or less complex set of causes that act together^6^. Therefore, there is no single model of explanatory factors that can be universally applied in different territories, at different times and in human groups that evolve with their own characteristics, even if they are influenced by global processes^7^.

These conceptual fundamentals are well illustrated when, as in the case of this study, the epidemiological behavior of diarrheas in children under five years of age in the state of Pernambuco is analyzed, in a relatively brief period of time, i.e., in the years 1997 and 2006. In a descriptive approach, marked geographical changes were observed in the prevalence of the problem, its spatial and socio-environmental distribution, its hospitalization demands and its participation (or impact) in the composition of the causes of death. It is a chain of events that, in addition to its own significance, as a representation of a specific nosology, is part of a more general process: the recent history of transition^18^.

It is clear both in terms of univariate analysis and in terms of understanding diarrhea under a multivariate approach, the significant differences that demarcate the epidemiological transit of the problem in a specific group of hosts: children under five years old.

From this perspective, the significant variations recorded in the five groups of factors and their subcategories, composed of three levels of multivariate analysis, stand out. The entry or exclusion of many variables by more statistical criteria than conceptual ones can, along with other limitations that will be opportunely highlighted, represent a possible impropriety of methodological approach. However, the prevailing impression is that diarrhea, as a healthmapping nosography, expresses in a sensitive way the dynamic process of a set of factors that are articulated from the structural level of society to the family or individual instance of its determination, which causes changes in analytical results in the period evaluated.

Based on the results of the univariate and multivariate analyzes treated in our study, we highlight two aspects (the notable reduction in the prevalence of diarrhea in the Metropolitan Region of Recife and the relationship that the water supply or treatment statistically meant in reducing the risk of this problem in children), it would be possible to presume two concordant ecological events. The first refers to the great advances in the coverage of public water supply in the Metropolitan Region of Recife over the last 10 years, unlike in the rest of the state, whether in urban or rural areas. The second refers to the water supply crisis that worsened in a significant way, so that the solution for several years consists in the construction of two large systems of water mains in the *Sertão* and *Agreste*, still in progress, for the populations of urban and rural areas through the transposition of the São Francisco river^19^ ^,^ ^20^. These observations are compatible with the explanatory hypothesis suggested here and would be two cartographies separated by the basic sanitation conditions (water, sewage, and garbage).

In Brazil, it is estimated that 36 million people still do not have access to safe drinking water. In addition, less than half of the Brazilian population does not have garbage collection and only 38% of the sewage is not treated, which contributed to the 212,000 hospitalizations in 2011 due to diarrhea in children under five years of age in the country^21^. A study carried out in Bahia to evaluate the impact of the *Água para Todos* (Water for All) Program, which included 224 municipalities, concluded that those with a coverage of 10% had a 14% reduction in mortality due to diarrhea in children under five years of age and in 6% in hospital admissions, when compared to uncovered municipalities or those with lower coverage^22^.

When analyzing the hierarchical final models of the study years, 1997 and 2006, we highlight some variables that have been associated with diarrhea in the two periods, such as maternal age, child age and number of people per room. In other situations, there are variables that were not included in the adjusted model, such as water supply and treatment in 1997, but which entered in 2006. On the other hand, some groups of variables that appear in the final model of 1997, such as the destination of garbage and the availability of refrigerator at home, which was part of the risk model in 1997, stopped participating in 2006.

The age of the mother has frequently been associated with the prevalence of diarrhea^23^ ^,^ ^24^. The association of the disease in the children of younger mothers can be attributed to the greater probability of conceiving underweight children, as well as maternal inexperience in caring for children and greater difficulties in adequately feeding their children, including those with a tendency towards early weaning^25^ ^,^ ^26^.

The age of the child implied a higher risk in the groups of zero to 11 months and 12 to 24 months, compared to the older children. This is a universal trend, with several biological, cultural and socio-environmental reasons for this increased risk^27^.

Despite the increase in income in the nine-year period (1997–2006), due to the increase in the minimum wage, income transfer program and the country's own economic development, in Pernambuco, there is still a considerable number of cases in which the *per capita* family income is below 0.5 (half) minimum wage, a condition that has played a significant role in the multivariate model. Low income favors the occurrence of predisposing or aggravating factors of diarrheal diseases due to the low level of education and precarious conditions of life, constituting the so-called poverty ecosystem^28^ ^,^ ^29^. As an example, the variable “number of people per room” suggests that the high crowding of people can favor the precarious conditions of hygiene and the contamination of food and water. Likewise, these adverse conditions apply to the treatment of drinking water, which came to play a significant role in the most recent analysis in 2006.

Other characteristics of dwellings, families, and children not highlighted in the simple or adjusted models are no longer debated here, but this does not mean that they cannot play a protective or risk role in other circumstances or context.

The study presents limitations that may affect in some respects its internal or external validity. The data were not generated from a study previously designed to analyze the risk factors of diarrhea. In addition, doubts about unexpected statistical results persist, as in the case of water treatment, without a convenient explanation, and there are also epidemiological limitations inherent in prevalence surveys with simultaneous cause and effect registries. Additionally, the adjustment of results as a function of the sample weight was not performed, since the initial study of the historical series (1991) did not contemplate these aspects in the state of Pernambuco^9^ or in any of the eight states of the Northeast surveyed; these are, therefore, restrictions that cannot be corrected.

Due to the comprehensiveness of factors evaluated, the originality of the approach, the temporal dimension of its trends and the epidemiological configuration of its observations and analyzes, this study can contribute to the proposal of political and programmatic alternatives to control the problem, as well as to motivate new surveys.

At the public policy level, despite changes in terms of people, time sequences, and geographic spaces, diarrhea remains on an important scale in the ranking of government power. Alongside these basic epidemiological variations, isolated or combined factors were identified in its determination, which signals new strategic indications for its effective control.
